# Contribution of serology in congenital toxoplasmosis diagnosis: results from a 10-year French retrospective study

**DOI:** 10.1128/jcm.00354-23

**Published:** 2023-09-20

**Authors:** Julie Denis, Jean-Philippe Lemoine, Coralie L'ollivier, Anne-Sophie Deleplancque, Hélène Fricker Hidalgo, Hervé Pelloux, Christelle Pomares, Bernard Cimon, Luc Paris, Sandrine Houzé, Isabelle Villena, Odile Villard

**Affiliations:** 1 Institut de Parasitologie et de Pathologie Tropicale, UR7292 Dynamique des interactions hôte pathogène, Fédération de Médecine Transrationnelle, Université de Strasbourg, Strasbourg, France; 2 Laboratoire de Parasitologie et Mycologie Médicale, Hôpitaux Universitaires de Strasbourg, Strasbourg, France; 3 Laboratoire de Parasitologie-Mycologie, CHU d'Angers, Angers, France; 4 Centre National de Référence Toxoplasmose-Pôle sérologie, Hôpitaux Universitaires de Strasbourg, Strasbourg, France; 5 IHU-Méditerranée Infection, Assistance Publique Hôpitaux de Marseille (AP-HM), Marseille, France; 6 Aix Marseille University, IRD, AP-HM, SSA, VITROME, IHU Méditerranée, Marseille, France; 7 CHU Lille, Parasitology Mycology Department, Lille University, Inserm, U995 - LIRIC - Lille Inflammation Research International Center, Lille, France; 8 Laboratory of Parasitology and Mycology, INSERM U1209, CNRS UMR5309, Grenoble-Alpes University Hospital, Institute for Advanced Biosciences, Grenoble Alpes University Hospital, Grenoble, France; 9 Parasitology-Mycology laboratory, Côte d'Azur University, INSERM 1065, Nice University Hospital, Nice, France; 10 Centre Méditerranéen de Médecine Moléculaire (C3M), U1065, Université Côte d'Azur, Inserm, Nice, France; 11 Angers University, Brest University, IRF, SFR 4208 ICAT, Angers, France; 12 Parasitology laboratory, AP-HP Pitié-Salpêtrière, Paris, France; 13 Parasitology laboratory, AP-HP, Hôpital Bichat - Claude Bernard, Paris, France; 14 University of Paris Cité, IRD 261, MERIT, Paris, France; 15 Department of Parasitology and Medical Mycology, National Reference Centre on Toxoplasmosis, Reims Hospital, Reims, France; 16 Team EA 7510, SFR CAP-SANTE, Reims Champagne Ardenne University, Reims, France; Mayo Clinic Minnesota, Rochester, Minnesota, USA

**Keywords:** congenital toxoplasmosis, serological diagnosis, immunoblot, IgM, IgA, CIP assay

## Abstract

This study aimed to evaluate different serological strategies for the postnatal diagnosis of congenital toxoplasmosis (CT) and establish a biological algorithm for CT diagnosis. The study analyzed serological data of immunoglobulins M, A, and G (IgM, IgA, IgG) performed by immunoenzymatic and compared immunological profile (CIP) assays in 668 newborns with CT diagnosis across four testing periods: P1 (D0– D10), P2 (D11–D35), P3 (D36–D45), and P4 (>D45). Forty-nine percent of the 668 CT cases were diagnosed during P1 and 34%, 4%, and 12% during P2, P3, and P4, respectively. CIP assays detected neosynthetized IgMs/IgGs in 98% of CT cases diagnosed during P1, while IgMs and IgAs were detected in 90% and 57% of CT cases diagnosed during P2 and in 88% and 67% of diagnoses made during P3, respectively. Detection of neosynthesized IgMs/IgGs, IgMs, and IgAs by immunoassay contributed to CT diagnosis in 81%, 77%, and 60% of cases, respectively. In total, 46% of serum samples were positive for all three parameters, 27% for two, and 27% for one of the three. The study recommends using the CIP assay as standard during P1 for CT diagnosis and IgM and IgA immunoassays after P1. A clinical and biological follow-up in a specialized center with a close collaboration between biologists and clinicians is highly recommended to increase the chances of early diagnosis. Overall, this study provides useful information for the development of a biological algorithm for CT diagnosis, which can aid in early detection and appropriate treatment of this disease.

## INTRODUCTION

Congenital toxoplasmosis (CT) is related to the transmission of *Toxoplasma gondii* to the fetus during pregnancy in cases of maternal primo infection during pregnancy (1).

Whenever there is a maternal toxoplasmic primo or an evolutive infection during pregnancy, CT must be investigated. Prenatal CT diagnosis is based on parasitic deoxyribonucleic acid detection in the amniotic fluid by using polymerase chain reaction (PCR)-based tests. Postnatal CT diagnosis can be established also by the same technique but performed on the umbilical cord blood, the newborn’s peripheral blood, or the amniotic fluid collected during childbirth. To confirm the CT diagnosis in case of negative results or the absence of PCR test, serology appears as the most efficient diagnosis method. The serological work-up includes (i) testing for neosynthesized anti-*Toxoplasma* immunoglobulin IgGs (during the first 3 months) and/or IgMs (only during the first month) using compared immunological profiles (CIP) assays, (ii) testing for specific IgMs and/or IgAs using immunoanalysis, and (iii) IgG kinetics work-up (2 – 6). In cases of confirmed CT, it is advised to start specific treatment as soon as possible after birth (5, 7) and clinical and ophthalmological follow-up are recommended (8).

Our objective in this study was to evaluate the efficacy and contribution of the different serological assays to CT diagnosis during the postnatal period when the kinetics alone of IgG does not allow to confirm the diagnosis of CT and to propose standardization of the management of newborns with suspected CT.

## MATERIALS AND METHODS

### Study design

This retrospective study analyzed 10 years’ (2007–2017) worth of newborn cases presenting a postnatal CT diagnosis in 23 French University Hospitals *via* the TOXOSURV network set up in 2006 by the French national toxoplasmosis reference center (*Centre National de Référence—CNR Toxoplasmose*) (9).

We collected clinical and biological data including serological results from the mothers and their infants at childbirth, as well as at different follow-up time points, along with data regarding the different serological techniques used. Serological data (IgMs/IgAs using immunoanalysis testing and neosynthetized IgMs/IgGs using CIP tests) from samples taken from birth were analyzed. Several assays were used to measure IgMs/IgAs depending on the laboratory (^1^, reagents validated in newborns; ^2^, IgM assay; ^3^, IgA assay): agglutination ISAGA (BioMerieux)^1,2,3^, Platelia (BioRad)^1,2,3^, VIDAS/VIDIA (BioMérieux)^2^, AxSYM/Architect (Abbott)^2^, ACCESS/DXI (Beckman Coulter)^2^, Elecsys Cobas (Roche Diagnostic)^2^, Liaison (Diasorin)^2^, and Immulite (Siemens)^2^. CIP assays were performed to detect neosynthetized IgMs/IgGs using *Toxoplasma* Western Blot IgG-IgM (LDBIO) or an enzyme-linked immuno-filtration assay technique (10).

### Serological parameters studied

The efficacy of IgMs/IgAs detection assays and CIP techniques was analyzed based on the sample that led to the CT diagnosis, depending on the time period in which the analysis was performed. Four periods were defined: the P1 period (10 first days of life of the newborn), the P2 period (11th–35th day), the P3 period (36th–45th day), and the P4 period (46th–90th day).

### Exclusion criteria

Analysis reports were excluded from the study when the following information was missing: age of the newborn at sampling time, type of serological assay, or in the case of incomplete serological results. Cases diagnosed antenatally were excluded. Analysis reports in which the neonatal molecular biological diagnosis was positive were also excluded if no sera were collected at the same time as initiated treatments could have interfered with the serological results (11, 12). Finally, the reports of newborns whose CT diagnosis had been established solely based on IgG kinetics using immunoenzymatic techniques were also excluded because they did not comply with the four periods defined. Finally, sera sampled after CT diagnoses were excluded from the analysis of the contribution of each parameter (positive rates and Venn analysis) regardless of the periods as antiparasitic treatment could interfere with the following serological results.

## RESULTS

### Study population

**Fig 1:**
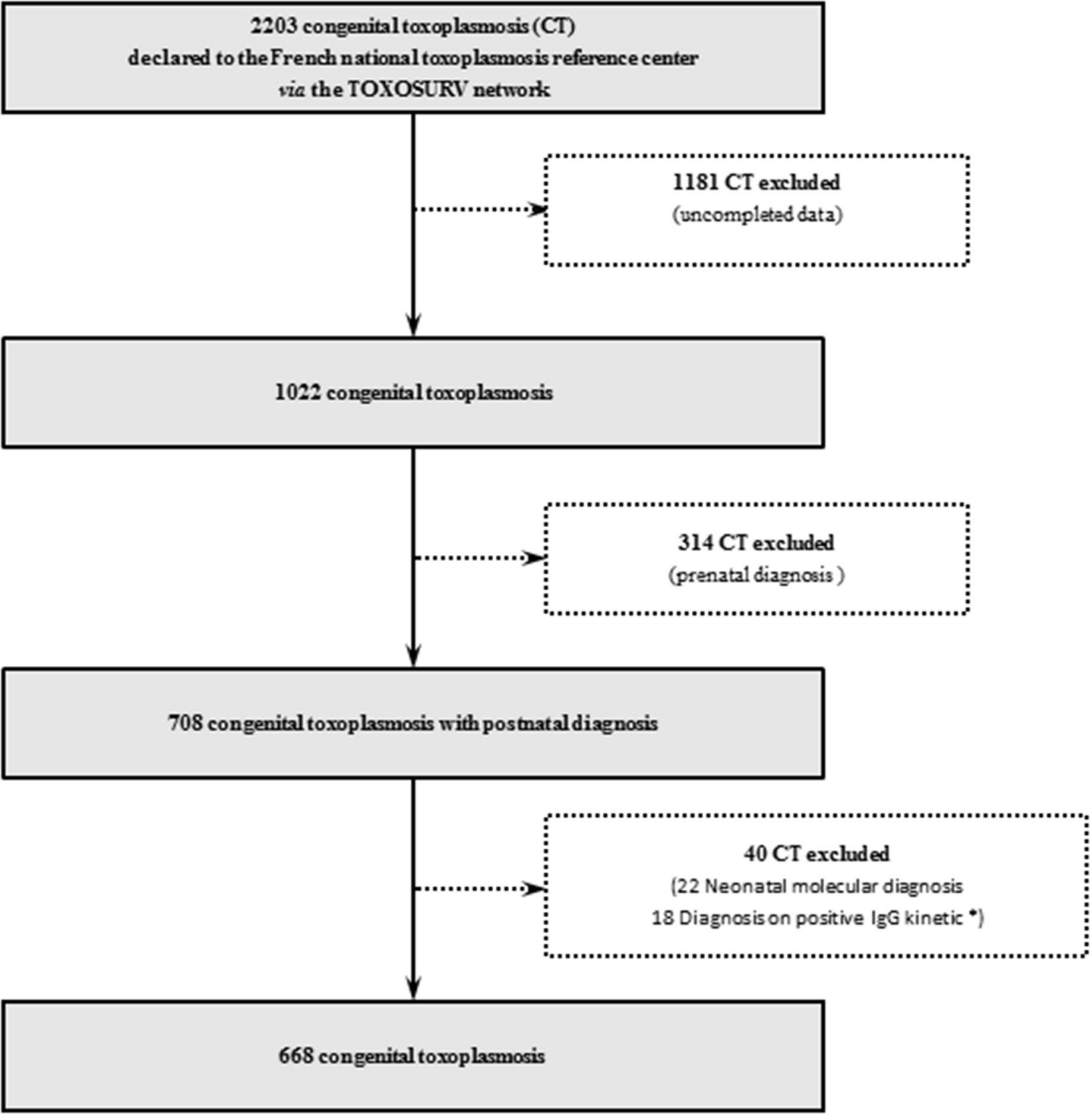
Study design. *We noticed 18 late CT diagnoses:10/18 with no CIP, IgM and IgA serological monitoring according to good practice (negative serology on P1 with no control before D90), and 8/18 with a correct but negative serological follow-up.

A total of 2,203 CT records were collected during 10 years from 27 centers (all experts in toxoplasmosis diagnosis), 1,022 of which were complete. Among these, 354 were excluded. Overall, 668 records of CT from 23 centers were analyzed, consisting of 2,525 serum samples (Fig. 1).

### Serological diagnosis of CT

The serological work-up included IgM/IgA detection by immunoanalysis and investigation of any neosynthesized IgMs/IgGs using CIP assay. *Toxoplasma* testing was performed by all centers for IgMs and by 74% of the centers for IgAs. IgM and IgA were measured on the first three samples in 98% and 88% of cases, respectively. Reagents validated for newborns were used in 87% and 70% of assays, respectively.

Our analysis of the centers’ different practices demonstrated that 87% of laboratories performed CIP assays to detect neosynthesized IgMs and/or IgGs. The test was performed on the first serum sample (S1) in only 62% (414/668) of cases. Depending on the center, we noted four strategies for CIP tests realization: (i) one test performed at birth; (ii) one performed during P1 with a control test performed during P2 when necessary; (iii) one performed at birth with control tests performed during P2 and P3; (iv) only one CIP test performed during P1 or P2.

### Practices analysis

On average, four serum samples (range: 1–21) were drawn from each of the 668 newborns during their follow-up. The first (S1) and second samples (S2) were taken during P1 in 87% and 61% of cases, respectively. Of note, 77% of S1 samples were collected within the first 3 days of life. The median follow-up time between S1 and S2 was 14 days compared to 30 days between S2 and S3. We also noticed one or more control serum samples were collected in 73% of patients once the CT diagnosis was established.

### CT diagnosis

**Fig 2:**
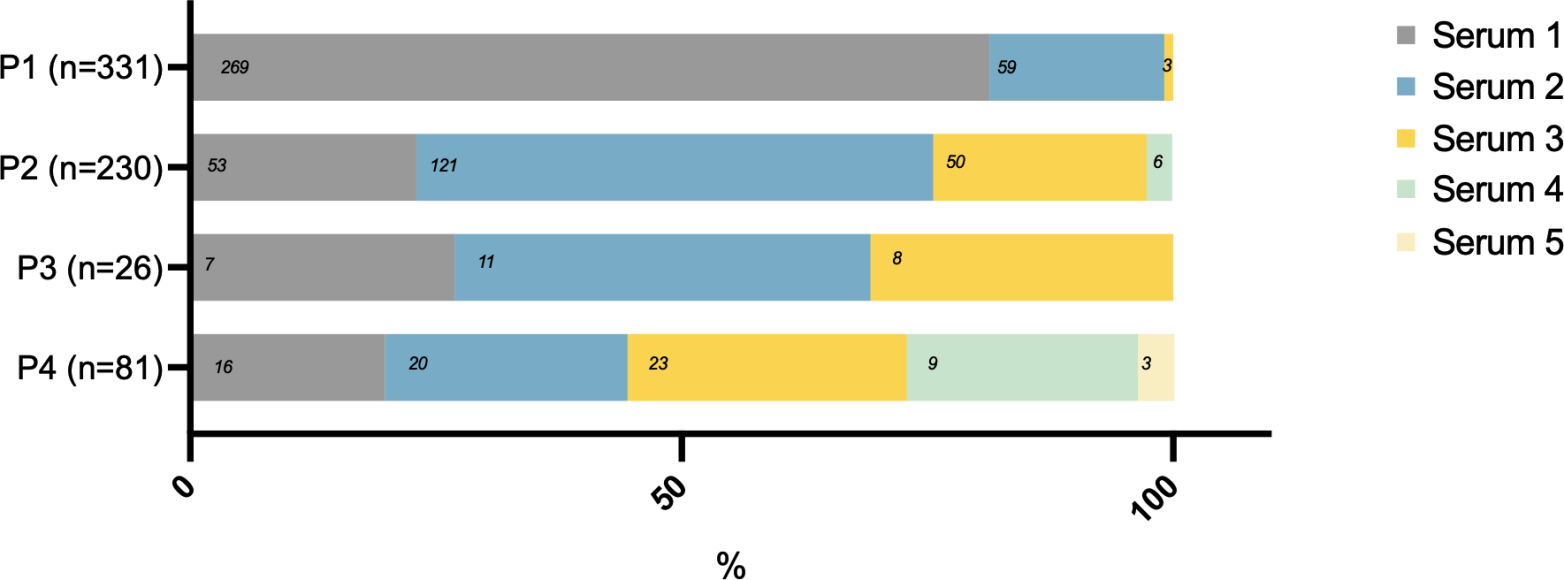
Categorization of the serum samples that led to CT diagnosis according to the period. CT, congenital toxoplasmosis; P, period (P1 = D0–D10; P2 = D11–D35; P3 = D36–D45; P4 = D46–D90).

CT diagnosis was established in 83% (555/668) of cases during P1 or P2 based on one of the three first serum samples taken: 48% (322/668) on S1, 27% (180/668) on S2, and 8% (53/668) on S3 (Fig. 2).

CT was diagnosed late during P4 in 12% (81/668) of cases. For 2% (16/668), S1 was taken during P4. In 10% (65/668) of newborns, S1 drawn during P1 was negative (for neosynthetized IgMs/IgGs and IgAs/IgMs detection) and the serum that led to the diagnosis was taken at P4 with a median time of 60 d after S1.

Finally, 6% (40/668) of CT diagnoses were established based on IgMs and/or IgAs detection during P1 with no serological confirmation of the positive result thereafter.

### Participation of the three parameters to CT diagnosis

#### P1 period

**Fig 3:**
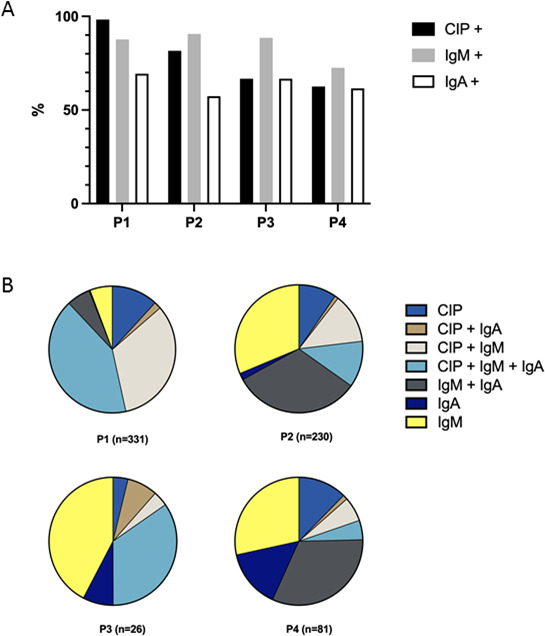
(A) Positivity rates of the total of compared immunological profile, IgMs, and IgA on sera that led to the CT diagnosis (positive sera/number of sera for which the technique was performed) immunoanalyses. (B) Proportions of positive techniques by combination on the sera that led to the CT diagnosis. Tests were considered positive or negative/not realized. P, period (P1 = D0–D10, *n* = 331; P2 = D11–D35, *n* = 230; P3 = D36–D45, *n* = 26; P4 = D46–D90, *n* = 81).

CT diagnosis was established during P1 for 49% (331/668) of newborns. Over this period, 98% (325/331) of serum samples benefited from an IgM test, 71% (235/331) from an IgA test, and 89% (296/331) from a CIP test. When a CIP assay was performed, neosynthetized IgGs/IgMs were detected for 98% (291/296) of CT cases. When IgM assays were performed, results were positive in 88% (285/325) of cases, compared to 69% (163/235) of cases with positive IgA tests (Fig. 3A). In 12% (39/331) of cases, CIP assay was the only positive test. Presence of IgMs and/or IgAs led to CT diagnosis in 12% (40/331) of cases when the CIP test was negative (positive IgM and/or IgA tests confirmed during P1) (Fig. 3B).

#### P2 period

For 34% (230/668) of CT cases, the diagnosis was established during P2. A CIP test was performed in 43% (98/230) of cases enabling the detection of neosynthesized antibodies in 82% (80/98) of cases. T IgM and/or IgA tests were performed in 97% (223/230) and 80% (183/230) of patients, respectively, with positive results in 91% (202/223) of cases for IgMs and in 57% (105/183) for IgAs (Fig. 3A). During this period, each patient benefited from an IgM and/or IgA test with a positive result for 90% (208/230) of them. IgM and/or IgA tests allowed for 65% (150/230) of CT diagnoses during P2 in patients with a negative or absence of CIP test (Fig. 3B).

#### P3 period

During this period, 4% (26/668) of CT cases were diagnosed. All serum samples were tested for IgMs versus 81% (21/26) for IgAs and 23% (6/26) for CIP assay. IgM and IgA testing were positive in 88% (23/26) and 67% (14/21) of cases, respectively, as was CIP assay (4/6) (Fig. 3).

#### P4 period

During P4, 12% (81/668) of CT cases were diagnosed. IgM serological tests were performed for 99% (80/81) of patients during this period, yielding positive results in 72% (58/80) of the cases. IgA tests were performed for 80% (65/81) of patients, with a positivity rate of 62% (40/65). During P4, 40% (32/81) of serological tests included CIP assays, which had a positivity rate of 63% (20/32) (Fig. 3).

### Diagnostic contribution of each assay

**Fig 4:**
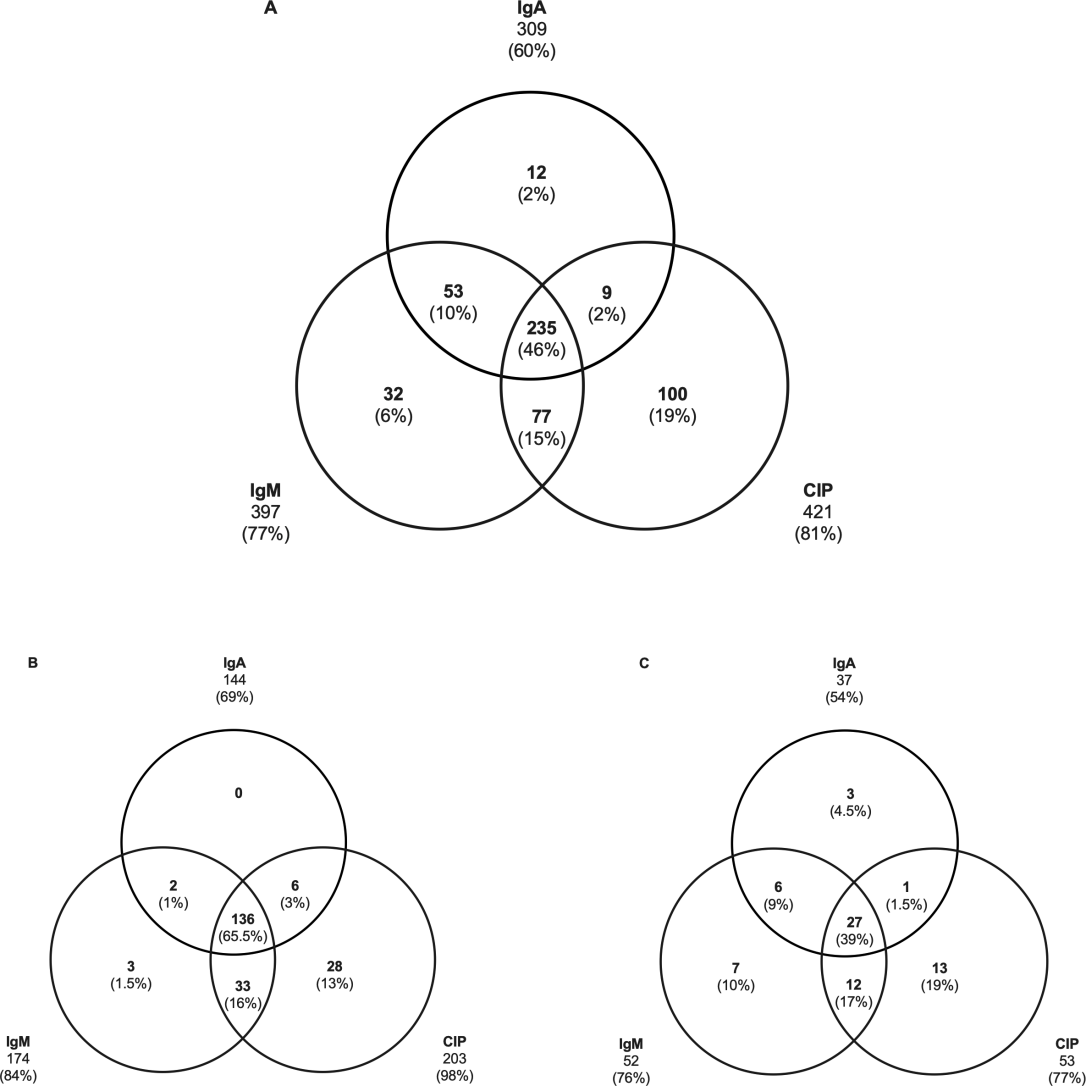
Venn diagram. Contributions of the CIP assays, IgM, and IgA serological tests to CT diagnosis. (A) For the four periods (*n* = 299 CT, i.e., 518 serum samples). (B) For the P1 period (*n* = 208 CT, i.e., 208 serum samples). (C) For the P2 period (*n* = 69 CT, i.e., 69 serum samples). Ig, immunoglobulin.

All three assays (CIP, IgM tests, and IgA tests) were performed in parallel on 21% (518/2,525) of the samples, representing 299 CT cases and enabling us to compare their respective contributions to the CT detection. CIP assays allowed the diagnosis in 81% (421/518), IgM tests in 77% (397/518), and IgA tests in 60% (309/518) of cases. Only 45% (235/518) of samples tested positive with all three techniques, while 27% (139/518) were positive for two (15% CIP and IgM tests, 10% IgM and IgA tests, 2% CIP and IgA tests). In 28% (144/518) of cases, only one assay was positive (19% for CIP, 6% for IgM tests, 2% for IgA tests) (Fig. 4A). During P1, all three assays were performed in 208 CT cases. CIP assays were positive for 98% (203/208) of them (Fig. 4B). For the 69 CT diagnosed during P2, CIP tests were positive for 77% (53/69) samples compared to 76% (52/69) and 54% (37/69) for IgM and IgA tests, respectively (Fig. 4C).

## DISCUSSION

### Follow-up frequency

Our study enabled us to show the wide variety in how different expert centers operate in terms of follow-up frequency during a newborn’s first 3 months of life. Follow-up was most often structured based on the protocols established in each center. A majority of newborns (87%) were provided with at least one serological screening work-up during P1, and half of CT cases were diagnosed during this time. This early diagnosis is important because it allows for rapid treatment implementation. The sample frequency varied quite widely across the centers. Our study revealed that CT diagnosis was established during the first month of life for the majority of infants (83%), thus emphasizing how important it is to implement close neonatal and postnatal follow-ups. We also noted that 12% of CT cases were diagnosed late, i.e., during P4. For all of these cases, newborns had been lost to follow-up after being discharged and did not benefit from early serological work-up, hence the late diagnosis. By performing an initial sampling during the P1 period and a work-up during the following month, a majority of CT cases can, thus, be diagnosed. Furthermore, late diagnoses can be reduced by strengthening cohesion between laboratories, obstetric-gynecological and pediatric departments, and implementing postnatal diagnosis protocols.

### Test efficacy and CT diagnostic contribution

For the centers where the CIP technique is available, this test is systematically carried out during the first month of life. However, a wide variety of strategies have been observed regarding the infant’s age at the time of the first test and the frequency with which this test was performed.

CIP test greatly contributed diagnosis, providing positive results in 81% of cases and representing the only positive parameter in 19% of cases. Its value was found to be especially high during P1, with a positivity rate of 98%. When comparing infected and not infected population, previous study reported positivity rates from 33% to 79% (10, 13 – 15). During this period, it was the only parameter able to confirm CT diagnosis by revealing neosynthesized IgMs/IgGs (13 – 15). The positive immunoenzymatic tests from this period, for example, did not enable immunoglobulin originating from the mother to be distinguished from those synthesized by the newborn.

All the laboratory centers measured IgM levels and, in 87% of cases, they did so by using a technique validated for use in newborns under 1-year old (Toxo-ISAGA, BioMérieux and PlateliaTM Toxo, BioRad) (10). Performances for new CT diagnosis were identical when comparing P1 and P2 (88% and 91%, respectively). It was, on average, comparable to the sensitivity rates reported in previous studies that compared eperformances on infected and not infected populations (76.9%–100%) (10, 16, 17,18,19). Moreover, 74% of the laboratories measured IgA levels, and in 70% of cases, they used a technique that had been validated for use in newborns. Positivity rates were almost identical when comparing P1 and P2 (69% and 80%, respectively), yet poorer than the sensitivity of the IgM tests (20). Nevertheless, during P1, newborn serum can be contaminated with the mother’s serum, which could lead to false positive tests. Thus, IgM and IgA tests must be interpreted with precaution during this period.

During P2, CT diagnosis was established based on IgM and/or IgA levels in 65% of cases, highlighting the importance of these analyses during this time period. We noted the same results for P3 and P4 for patients with a correct serological follow-up (data not shown) which supports the continuation of serological screening despite several previous negative tests. In the postnatal diagnosis of CT, IgA measurement techniques were less sensitive and contributed less to CT diagnosis than IgM and CIP tests, with rates of diagnosis contribution of 60%, 77%, and 81%, respectively (irrespective of time period). However, testing for IgAs was found to solely be positive in 2% of the samples for which the other two techniques (IgM tests and CIP) produced negative results. IgA testing can be a good complement to IgM and CIP tests, notably in cases of negative results from other work-ups (21).

We excluded 18 CT cases from the analysis due to a diagnosis made solely by IgG kinetics. For 8 of them, the serological follow-up was correct, but the tests were negative highlighting the risk of false negatives for these techniques. However, only 1–2 tests were performed on these cases, emphasizing the complementarity of these tests.

This study evaluated the contribution of the different serological parameters for CT diagnosis on infants with no positive antenatal screening. It is the first to include such a large number of CT cases allowing, thus, the determination of the most suitable paired serological parameters according to the post-natal periods. We could not evaluate formerly sensitivity and specificity as only infants with a CT diagnosis were included, yet we observed high positivity rates of each parameter. These good performances could be explained by a short exposure to maternal treatment as most infants included were contaminated during the third trimester of pregnancy. So, these results could, therefore, be particularly interesting in suspected CT when prenatal diagnosis was not performed.

### Conclusion

**Fig 5:**
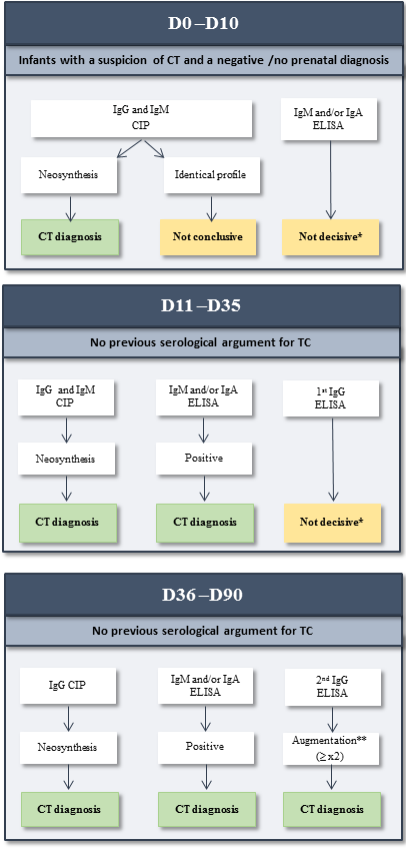
Recommandations from National Reference Center to CT serological diagnosis on an infant with negative or no prenatal diagnosis. *Risk of contamination by maternal immunoglobulins. **IgG must be measured in parallel on successive sera with the same technique.

This study enabled the CNR expert group to propose recommendations for postnatal CT diagnosis in case of negative or not performed prenatal diagnosis (Fig. 5).

Between day 0 and day 10, the CIP assay to detect IgM/IgG neosynthesis contributed the most to the CT diagnosis (98%) and should be standard for CT diagnosis. After day 10, testing for IgM and IgA is recommended and may be associated with CIP tests to detect the neosynthesis of IgMs/IgGs as well as testing for IgGs, which must be monitored monthly. Given the complexity of serological diagnosis, the expert group recommends clinical and biological follow-ups in a reference center, with collaboration between biologists, obstetricians, and pediatricians. This is of utmost importance to set up a close follow-up of the newborn whose mother seroconverted during pregnancy in order to detect and treat as soon as possible the CT.
